# Chronic Inflammation Impairs Male Fertility—A Case-Control Study in Ulcerative Colitis Patients

**DOI:** 10.3390/jcm10071460

**Published:** 2021-04-02

**Authors:** Artur Wdowiak, Mariusz Gujski, Iwona Bojar, Dorota Raczkiewicz, Joanna Bartosińska, Anita Wdowiak-Filip, Rafał Filip

**Affiliations:** 1 Diagnostic Techniques Unit, Medical University of Lublin, 20-081 Lublin, Poland; wdowiakartur@gmail.com; 2 Department of Public Health, Medical University of Warsaw, 02-097 Warsaw, Poland; mariusz.gujski@doktor.pl; 3 Department of Women’s Health, Institute of Rural Health in Lublin, 20-090 Lublin, Poland; iwonabojar75@gmail.com; 4 Department of Medical Statistics, Center of Postgraduate Medical Education, School of Public Health, 01-813 Warsaw, Poland; dorota.bartosinska@gmail.com; 5 Department of Cosmetology and Aesthetic Medicine, Medical University of Lublin, 20-093 Lublin, Poland; anita.wdowiak@gmail.com; 6 Department of Gastroenterology with IBD Unit of Clinical Hospital 2 in Rzeszow, 35-301 Rzeszow, Poland; r.s.filip@wp.pl; 7 Faculty of Medicine, University of Rzeszow, 35-310 Rzeszow, Poland

**Keywords:** inflammatory bowel disease, ulcerative colitis, semen analysis, antisperm antibodies, oxidation-reduction potential, male sex hormones, bacterial infection

## Abstract

Recent evidence indicates that a systemic state of inflammation may exert a negative effect on male fertility. The aim of this study is to evaluate sperm quality parameters in male patients with ulcerative colitis (UC). Between December 2019 and December 2020 semen analyses are performed in 50 patients with UC in clinical remission. The control group consists of 50 healthy volunteers. Total sperm count, sperm count, percentage of morphologically normal spermatozoa, viability, and progressive motility, are significantly lower in the study group than in healthy males (*p* < 0.001). The DNA fragmentation index (DFI) and oxidation-reduction potential (ORP) are significantly higher in the study group (28.9% and 1.55% on average, respectively) than in healthy males (14.6% and 0.79% on average, respectively). Bacteriospermia is more clearly observed in the study group (*p* = 0.037), and the most frequent pathogen is *Enterococcus faecalis*. The DFI and ORP are significantly higher in bacteria carriers, compared to males without microbial pathogens from both the study and control groups (*p* < 0.001). To conclude, UC patients have worse basic sperm parameters compared to their healthy counterparts. Deterioration of semen parameters, as well as an intensified DNA fragmentation could be a result of oxidative stress intensification.

## 1. Introduction

Inflammatory bowel diseases (IBD) have emerged as global diseases. Both ulcerative colitis (UC) and Crohns disease (CD) affect over two million individuals in North America, three point two million in Europe, and millions more worldwide. These data indicate that in recent years, IBD has become a global disease, with accelerating incidence in newly developed countries where lifestyle has become more westernized. Although the incidence is stabilizing in western countries, the burden remains high as prevalence surpasses 0–3% [1,2]. Although diagnosis can be made at any age from 0 to >90 years, in the vast majority of the populations patients with CD and UC are usually diagnosed in their 20s and 30s [3]. Therefore, both sexual health and fertility are serious concerns among many patients with IBD, and adequate knowledge of causative factors is of great importance.

Infertility is defined as the inability to conceive within 12 months of unprotected sexual intercourse [4]. Although there are data on the influence of selected medications, surgery, and nutritional disturbances on the sexual status in IBD patients, good quality data specifically pertaining to factors affecting male fertility in IBD are still lacking [5]. However, it should be pointed out that male infertility is considered to be more prevalent in IBD patients than in the general population [6,7].

Available data indicate that 5-aminosalicylic acid (5-ASA) and methotrexate (MTX) should be discontinued before conception. It was shown in UC patients that long-term 5-ASA administration may cause abnormal semen parameters, including impaired sperm cell motility, increased abnormal sperm morphology, as well as reduced sperm concentration [8,9,10]. MTX is used often as a second-line immunomodulatory agent in patients with IBD who are thiopurine-resistant or -intolerant and it is known to induce MTX oligospermia due to its anti-folate actions, with a consequent inhibition of DNA synthesis and cell proliferation [11,12]. A recent study by Ley et al. showed a significantly decreased sperm integrity secondary to oxidative stress, compared with age-matched men in IBD patients who received MTX for >three months [13]. The role of other medications including azathioprine (AZA) and 6-mercaptopurine (6MP), as well as steroids, has not been clearly established yet. However, no study has indicated significant adverse effects on male fertility nor on pregnancy outcomes [4,7,14]. Similarly, single reports arising from clinical trials with biological agents indicate that, at least with regard to those most prescribed, they did not cause adverse pregnancy outcomes and male infertility [15,16,17,18].

Disease activity via increased concentrations of pro-inflammatory cytokines, such as tumor necrosis factor (TNF) and interleukin (IL)-1, may increase cytokine-mediated anti-fertility effects. Moreover, active inflammation is accompanied by high levels of reactive oxygen species and oxidative stress, which may exert a negative effect on semen parameters [19,20,21]. Although there are no specifically addressed studies, it is known that a poor nutritional status with zinc deficiency also may add to infertility in men with IBD manifested e.g., by oligospermia [22,23]. Research data also indicate an association between tobacco smoking and alcohol consumption and male fertility, especially with regard to sperm count and testicular atrophy [24]. However, the direct relevance in IBD patients has not been elucidated yet. Abdominal pain, diarrhea, fatigue, as well as depression, are stressing factors which may significantly decrease the quality of life in IBD patients. Several reports have shown an association between stressful life events and poor semen quality, which may be attributed to hormonal disturbances [25].

Last but not least, immunological factors associated with humoral immunity against sperm also may lead to infertility. The underlying mechanism is the induction of antisperm antibodies (ASA) due to the cross-reactivity between antigens of spermatozoa and exogenous antigens, such as bacteria. During the course of IBD, intestinal permeability increases, which may lead to immunization against the gut microbiome, which shares common epitopes with human spermatozoa [26,27].

The aim of our study is to evaluate sperm quality parameters, including the presence of antisperm antibodies, in male patients with ulcerative colitis.

## 2. Materials and Methods

### 2.1. Study Group

Semen analyses were performed for 50 patients with unconfirmed fertility with ulcerative colitis (UC) inflammatory bowel disease (IBD) who were in clinical remission for at least a 3-month period. All subjects were enrolled from an Outpatients Gastroenterology Clinic, using a randomization list drawn up separately for this center between December 2019 and December 2020.

Inclusion criteria were as follows: Patients eligible for the study were aged 20–30 years at screening and had a confirmed diagnosis of UC with endoscopic and histopathologic evidence of UC for at least 12 months before enrolment. Patients had an inactive UC (Mayo endoscopy subscore = 0 or 1, rectal bleeding subscore = 0, stool frequency subscore = 0, physician’s global assessment subscore = 0) (and were on a UC drug holiday for 12 weeks).

Exclusion criteria were as follows: Patients were excluded if they used any UC therapy, including 5-aminosalicylic acids, azathioprine, 6-mercaptopurine, methotrexate, prednisone, budesonide, and biologics, in the 12 weeks before the study entry date. Those who had a previous UC diagnosis, documented problems with male reproductive health—such as a low sperm count, impotence, or malignancy—were not eligible for the study. Patients also were excluded if they presented clinical symptoms of active disease or had biochemical (C-reactive protein or calprotectin), endoscopic, or histologic signs of disease exacerbation. The use of other concomitant medications or dietary supplements which could interfere with semen parameters, as well as cigarette and alcohol consumption, was prohibited for a period of 12 weeks before study entry.

The Control group comprised 50 healthy men with unconfirmed fertility aged 20–30 years who did not use concomitant medications or dietary supplements which could interfere with semen parameters. They were recruited through advertisements posted on the internet.

Following study enrolment, patient data that focused on male health issues, fertility, and IBD history including medications, were collected. Basic clinical evaluation, followed by laboratory and endoscopy result assessments, were performed by a gastroenterology consultant.

Body mass index (BMI) was calculated: Normal weight 18.5–24.9 or overweight 25–29.9 kg/m^2^.

All participants gave their informed consent to participate in the study. The study was financed by the Medical University in Lublin and approved by the Bioethical Committee at the Medical University in Lublin No. KE-0254/324/2019.

### 2.2. Semen Samples

Semen was collected by masturbation after 3–5 days of sexual abstinence and examined with a computer-assisted semen analysis (CASA) system according to WHO 2010 guidelines [28].

The direct mixed antiglobulin reaction (MAR) test was carried out as an immunological screening assay for antibodies bound to the sperm using a direct immunoglobulin G (IgG)-MAR test (SpermMAR, FertiPro N.V., Beernem, Belgium). Approximately 10 μL of fresh semen was placed onto a glass microscope slide, mixed with 10 μL of the IgG suspension and, subsequently, an anti-IgG incomplete antibody was added as a bridging element. The drop was covered with a cover glass and incubated for 10 min. Then, the percentage of motile sperm bound to beads and the predominating attachment regions were determined (head, tail, tail tip) by means of a phase-contrast microscope at a 400× magnification. When the majority of sperm cells showed attached beads on the head, as well as along the tail, the pattern was considered as ‘mixed’. All IgG-positive samples were further tested for IgA-ASA (antisperm antibodies) (≥10%) by means of IgASperm MAR (FertiPro N.V., Beernem, Belgium) in accordance with the manufacturer’s instructions [28].

The oxidation-reduction potential (ORP) in human semen and seminal plasma were measured using the Male Infertility Oxidative System MiOXSYS (Aytu Bioscience, Englewood, CO, USA), the technology based on a galvanic measure of electrons. To begin the procedure, disposable test sensors, each with a built-in three electrode system, were inserted into a galvanostatic analyzer. Following the application of the sample onto the sample port, it finally reached the reference electrode, completed the electrochemical circuit, and signaled the analyzer to emit a low voltage oxidizing current between electrodes to generate the ORP. The displayed ORP measurement reflected the average of the final ten seconds, and the ORP value was displayed on the analyzer screen as millivolts (mV) [29].

The percentage of fragmented DNA in spermatozoa was determined using the sperm chromatin dispersion test, in accordance with the instructions provided by the manufacturer (Dyn-Halosperm Kit; Halotech DNA SL, Madrid, Spain) [30]. Sperm immersed in the agarose medium was exposed to an acidic solution and, subsequently, a lysing solution. It was observed that spermatozoa with fragmented DNA had a small or no ‘halo’, whereas non-fragmented DNA showed big halos of the dispersed DNA loops. Then, 300 cells were counted per sample, and the percentage of sperm with DNA damage calculated.

Patients were instructed to perform semen collection after careful genital hygiene and discard of the first release of urine. All samples were transported for analysis to the laboratory of clinical microbiology.

### 2.3. Detection and Identification of Cultivable Pathogens

Standard bacteriological culture methods were used to detect genital pathogens from semen specimens. Semen was used directly upon arrival at the laboratory. Selective and differential solid media (GRASO, Starogard Gdański, Poland) were used for cultivable microorganisms, including gram-positive cocci, gram-negative bacteria, lactobacilli, anaerobes, and fungi. Species identification was carried out using Matrix for Matrix-Assisted Laser Desorption/Ionization-Time of Flight-Mass Spectrometry (MALDI-TOF-MS) measurements in the microorganism identification Bruker MALDI Biotyper Compass 4.1 (BRUKER, Bremen, Germany) coupled with Applied MSP Library(ies): BDAL/contains 8468 MSPs/dee78ccc-c113-4ede-b02e-392d0cde98c1/2019-12-12T16:22:11.571 with a cut-off identification ≥ 99%. Semen samples were considered positive if bacterial concentrations were ≥5 × 103 cfu/mL, according to WHO guidelines recommending 10^3^ cfu/mL as the cut-off value for ‘significant bacteriospermia’ [28,31].

Regarding the study group, *Enterococcus faecalis* were found in 8 males, *Streptococcus agalactiae* in 2, *Escherichia coli* in 3, while in the control group—*E. faecalis* in 3, *S. agalactiae* in 1, and *E. coli* in 1 male.

A morning blood sample (5 mL volume) was obtained and sent to an authorized laboratory to assess serum levels of testosterone, lueinizing hormone (LH) and follicle-stimulating hormone (FSH).

### 2.4. Statistical Methods

The data were analyzed using STATISTICA 13 software, Statsoft, Tulsa, OK, USA. The mean (M) and standard deviation (SD) were estimated for numerical variables, as well as absolute numbers (n) and percentages (%) of the occurrence of items for categorical variables.

The following statistical tests were used:

- Chi-square test to compare categorical variables between the study group and the control group;
- Student’s *t*-test to compare numerical variables between the study group and the control group. Due to the large sample sizes we assumed that the parameter estimators were asymptotically normally distributed due to the central limit theorem, so parametric tests were used;
- Pearson’s correlation coefficient r to correlate numerical variables between each other. All correlations were estimated separately in the study group and separately in the control group;
- Fisher’s exact test to correlate presence of anti-sperm antibodies with abnormal semen parameters;
- Mann-Whitney’s U test to compare semen parameters, oxidation-reduction potential ORP, and DNA fragmentation index (DFI) between males with and without microbial pathogens. All comparisons were conducted separately in the study group and separately in the control group. Due to the small sizes of some subgroups, non-parametric tests were used.

The significance level was assumed to be 0.05 in all statistical tests.

## 3. Results

### 3.1. Characteristics of the Study Group and the Control Group

All males from the study group had a normal body weight with a median of 22.5 kg/m^2^ and an interquartile range of 20.6–23.2 kg/m^2^, whereas in the control group the median body mass index (BMI) was 23.1 kg/m^2^ with an interquartile range of 21.7–26.0 kg/m^2^, and 26% were overweight.

The largest number of the examined males in both groups had a secondary school education (60% in the study group and 58% in the control group), performed intellectual-physical work (66% each in both groups), and an average income per person in a household (60% and 58%, respectively). Approximately 1/3 of the examined males in each group lived in a city, the same percentage in a town, and the same percentage in a rural area (Table 1).

### 3.2. Comparison of Semen Parameters and Sex Hormones between the Study Group and the Control Group

Total sperm count, sperm count (mln/mL), percentage of morphologically normal spermatozoa, viability, and in progressive motility were significantly lower in the study group than in healthy males (*p* < 0.05 for all above-mentioned parameters). The DNA fragmentation index (DFI) and oxidation-reduction potential (ORP) were significantly higher in the study group (28.9% and 1.55% on average, respectively) than in healthy males (14.6% and 0.79% on average, respectively). A higher percentage of males from the study group (26%) had a microbial pathogen, compared to the control group (10%), (*p* = 0.037). Semen volume and concentrations of the examined sex hormones did not differ significantly between the study group and the control group (Table 2).

**Figure 1:**
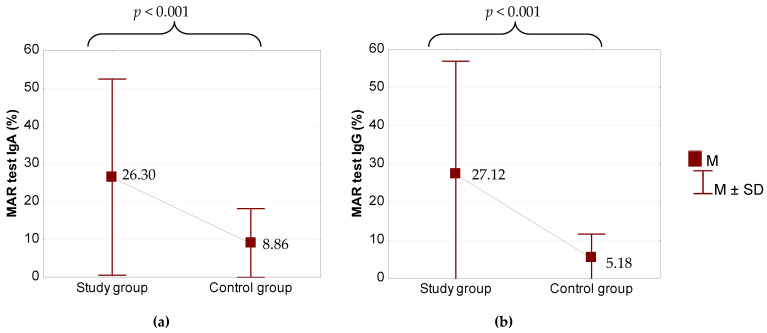
Comparison of MAR IgA (**a**) and IgG (**b**) between the study group and the control group. *p* for Student’s *t*-test. M—Mean, SD—Standard deviation, MAR—mixed antiglobulin reaction, Ig—immunoglobulin

A significantly higher percentage of anti-sperm antibodies was found in the study group than in the control group, screened by means of a MAR test, for both IgA (*p* < 0.001) and IgG (*p* < 0.001) (Figure 1).

### 3.3. Correlations of Semen Parameters with Anti-Sperm Antibodies, DNA Fragmentation Index (DFI) and Oxidation-Reduction Potential (ORP) in the Study Group and the Control Group

Regarding both groups in the study, the percentage of the MAR test for IgA and IgG did not correlate with a total sperm count, sperm count (mln/mL), semen volume, percentage of morphologically normal spermatozoa, or viable and in progressive motility (Table 3).

Both in the group of males with inflammatory bowel diseases and those who were healthy, the DNA fragmentation index (DFI) correlated negatively with the total sperm count, sperm count (mln/mL), percentage of morphologically normal spermatozoa, viable and in progressive motility.

Both in the study group and in the control group, oxidation-reduction potential (ORP) correlated negatively with the total sperm count, sperm count (mln/mL), percentage of morphologically normal spermatozoa, viable and in progressive motility.

The DFI and ORP did not correlate with sperm volume in the study and control groups.

Semen parameters, DFI, and ORP did not correlate with body mass index (BMI) both in the study group and in the control group (*p* > 0.05).

No significant correlations were found between the presence of anti-sperm antibodies and abnormal semen parameters both in the study group and the control group (Table 4).

### 3.4. Semen Parameters, Oxidation-Reduction Potential (ORP) and DNA Fragmentation Index (DFI) Versus Microbial Pathogen

Both in the study group and the control group, sperm count (mln/mL), as well as the percentage of morphologically normal spermatozoa, viability, and progressive motility, were significantly lower in asymptomatic carriers of bacteria than in males with a negative microbial pathogen (Table 5).

The DNA fragmentation index (DFI) and oxidation-reduction potential (ORP) were significantly higher in asymptomatic carriers, compared to males with a negative microbial pathogen from both the study and control groups.

Regarding the study group, a significantly lower total sperm count was found in bacteria carriers than in non-carriers (*p* < 0.001), whereas in the control group no such correlation was found. No correlations were found between the bacteria carrier state and semen volume, neither in the study nor the control group.

A significant positive correlation was observed between the DFI and ORP. An increase in the ORP was accompanied by an increase in the DFI value, on average, in the study group (r = 0.933, *p* < 0.001), as well as in the control group (r = 0.976, *p* < 0.001).

## 4. Discussion

The results of the study showed that semen parameters in males with inflammatory bowel diseases were worse, compared to their healthy contemporaries. The relevant data from the literature are full of contradictions; however, the majority of the publications present the deterioration of reproductive functions in these males. The methodological problem in most reports are the relatively small groups of the examined males. Shin, T. et al. undertook an attempt to systematize knowledge concerning this issue, and their analysis is consistent with the results obtained in this study [7]. Moreover, in a study by Martin, L. et al. semen analysis values among males with ulcerative colitis (UC) or Crohns disease (CD) were not significantly impacted, compared to those who were population-based [32].

Currently, the discussion remains unresolved concerning the problem as to what extent the decreased semen parameters examined, according to the WHO 2010 criteria, decide about male fertility. According to many authors, the most important parameter deciding about the time which will elapse until achieving pregnancy is sperm density [33,34,35]. The results presented in international literature concerning this problem are inconsistent as to whether the percentage of spermatozoa with normal structure has a predictive value for the achievement of pregnancy while trying to become pregnant naturally and through assisted reproductive technology (ART) [36,37,38,39,40]. During a study by Wdowiak, A. et al., based on a logistic regression analysis, it was found that together with an increase in such variables as semen density, percentage of morphologically normal spermatozoa, and progressive motility in ejaculate, and the anti-mullerian hormone (AMH) level in female partners, the chance for achieving pregnancy increased, and these relationships were statistically significant. Regarding the case of a partner’s age, viability, mixed antiglobulin reaction (MAR) test for immunoglobulin (Ig)A and IgG, no relationship was observed with achieving pregnancy [41]. Summing up, considering the classic assessment of semen parameters, it cannot be unequivocally determined as to what extent a decrease in semen parameters would affect the reproductive ability of males with inflammatory bowel diseases.

However, while analyzing such parameters as oxidation-reduction potential (ORP), or sperm DNA integrity, the results obtained may be alarming, because the value of ORP exceeds the normal, and the DNA fragmentation index (DFI) is close to the upper limit of the normal value. A study by Agarwal, A. et al. demonstrates that the ORP cut-off value (1.34 mV/10^6^ sperm/mL) was able to differentiate specimens with abnormal semen parameters with 98.1% sensitivity, 40.6% specificity, a 94.7% positive predictive value, and a 66.6% negative predictive value [42]. When used as an adjunct to traditional semen analysis, ORP levels may help identify an altered functional status of spermatozoa caused by oxidative stress in cases of idiopathic male infertility and in male partners of couples suffering recurrent pregnancy loss.

According to Karabulut et al., an excessive ORP level results in a decrease in sperm concentration, total motility, and progressive motility, which is confirmed by the results of the presented study [43]. Similar results were obtained by Garcia-Segura, S. et al.; however, only with respect to sperm motility [44]. An unfavourable effect of oxidative stress on sperm DNA fragmentation observed in our study also was described by Muratori et al. [45]

It may be expected that intensification of DNA fragmentation, as well as a decrease in some semen parameters in the inflammatory bowel disease (IBD) group may result from disorders in the oxidation-reduction potential of semen (increase in ORP) [46]. Considering the available literature there are no reports concerning ORP in the semen of males with inflammatory bowel diseases, and the presented study is the first which describes these relationships. There is increasing evidence that oxidative stress plays a crucial role in the pathogenesis and progression of IBD. The mechanisms which underlie the etiology of IBD have not been precisely explained; however, it is widely acknowledged that many factors, such as genetic susceptibility, changes in intestinal epithelial cells (IECs), dysregulation of immune responses, intolerance to the microbiota, and environmental factors as sources for oxidative stress jointly contribute to the development of IBD.

Many enzymes, e.g., peroxidases, nicotinamide adenine dinucleotide phosphate (NADPH) oxidase (NOX), xanthine oxidase (XO), lipoxygenases (LOXs), glucose oxidase, myeloperoxidase (MPO), nitric oxide synthase (NOS), and cyclooxygenases (COXs), are engaged in the endogenous production of reactive oxygen species (ROS), acting as catalysts of chemical reactions. Mucosal NOXs, such as the NOX2 complex, NOX1, and dual oxidase 2 (DUOX2), have been considered as emerging risk factors for IBD, confirming that redox homeostasis imbalance is crucial for the pathogenesis of IBD. XO, an enzyme present in the intestinal mucosa, is an important source of ROS and is involved in injuries to the gastrointestinal tract, whereas the activity of MPO in inflamed intestinal mucosa in patients with ulcerative colitis (UC) contributes to the progression of malignant tumors [47]. The effects of NOSs are contradictory, they maintain gastric mucosal integrity, but also cause injuries in UC and peptic ulcers [48].

Conversely, it was found that in IBD patients the amount of antioxidants, including vitamin C and beta-carotene, is insufficient mainly due to the limitations in vegetable and fruit consumption. Minerals, such as zinc (Zn), copper (Cu), manganese (Mn), and iron (Fe) are important for some antioxidant enzymes, Zn, Cu or Mn are essential for superoxide dismutase (SOD) isoforms, while Fe is a cofactor for the antioxidant enzyme, catalase. Antioxidant substances found in fruits and vegetables also include flavonoids which inhibit XO and the enzymes involved in ROS generation, such as COX, LOX, glutatione S-transferase (GST), and NOX [49].

The genetic variability in antioxidant/biotransformation enzymes may change their activity and increase the risk of IBD. NAD(P)H:quinone oxidoreductase 1 (NQO1) C609T and SOD2 Ala-9Val polymorphisms, for instance, are associated with the risk of UC, GSTM1 or GSTT1 mutations with the progression of IBD, while in patients with UC abnormalities in the activity of GST M1 are related to an early onset and more severe symptoms of the disease.

The presented study demonstrated that the asymptomatic carrier state of bacteria, especially *Escherichia coli*, in the semen of males with IBD may be one of the causes of imbalance in the oxidation-reduction system. The asymptomatic carrier state of bacteria more frequently observed in the group with inflammatory bowel diseases is probably the subsequent factor which decreases male fertility through an unfavourable effect on semen parameters. This was confirmed in a study by Ricci, S. et al., who showed the negative impact of *Enterococcus faecalis* on sperm quality. Additionally, the researchers confirmed that the presence of *E. faecalis* in genital samples of patients treated for infertility is predictive for a negative outcome of IVF (in vitro fertilization). During their study, *E. faecalis* was most frequently found (24.1%), followed by other microbial species including *Streptococcus agalactiae* (15.9%), and *E. coli* (15.4%), which is consistent with our observations [50].

It is considered that some commensal bacteria are potential pathogenic factors in the development of IBD, including *E. coli* which was more frequently found in the semen of the examined males with IBD, *Saccharomyces cerevisiae*, and *Mycobacterium avium paratuberculosis* (MAP). Mucosa-associated *E. coli* was observed in 68% of patients with UC, compared to 6% of those in the control group, which suggests its potential pathogenic role [51]. Overproduction of the ROS in intestinal epithelial cells leads to dysbiosis. Direct ROS production by intestinal microbiota during inflammation may be the cause of DNA damage in infected cells [52,53].

Antisperm antibodies (ASA) exert many effects on various aspects of fertilization, including acrosome reaction, capacitation, fertilization, and implantation. Nevertheless, the evidence for these effects still remains insufficient. During our study, the effect of the presence of antibodies on the basic semen parameters, the DFI, or ORP was not observed. This is partly in accordance with a study by Zini et al., who also did not confirm the relationship between the presence of ASA and sperm DNA integrity or morphology. However, in their study sperm concentration and progressive motility were significantly lower in ASA-positive patients [54].

Regarding the group of males with inflammatory bowel diseases, antisperm antibodies were significantly more often found compared to their healthy contemporaries. Similar observations were reported by Dimitrova et al. and Rossato et al. [6,27]. The existence of enhanced humoral immunity against sperm antigens in patients with ulcerative colitis might be a result of the increased intestinal permeability and may be associated with immunization against antigens of the common intestinal flora possessing common antigenicity with spermatozoa.

The presented study demonstrated that among males with inflammatory bowel diseases there occurs an intensification of oxidative stress in sperm, which is probably the cause of the deterioration of its basic parameters and the unfavourable effect on DNA fragmentation. Intensification of oxidative stress may be associated with an asymptomatic carrier state of bacteria. However, etiopathogenesis of this phenomenon requires further studies on a larger group of males. Although, in the presented study, we have probably avoided possible biases related to disease activity/medication, we realize that in real life it is unlikely to see UC patients without medication or disease activity. Therefore, future studies should consider patients on different medications and also should pertain to the different clinical scenarios.

## 5. Conclusions

1. Males diagnosed with inflammatory bowel diseases have worse basic sperm parameters compared to those who are healthy.

2. Regarding the sperm of males ill with inflammatory bowel diseases, the phenomenon of oxidative stress is intensified, which may be the cause of the deterioration of semen parameters, as well as an intensified DNA fragmentation.

3. An asymptomatic carrier state of bacteria may contribute to the intensification of oxidative stress.

## Figures and Tables

**Table 1:** Characteristics of the study group and the control group.

Variable	IU or Category	Parameter	Study Group (N = 50)	Control Group (N = 50)	*p* ^1^
Age	Years	Min-Max, M ± SD	23–35, 28.9 ± 3.6	24–36, 29.5 ± 3.9	0.426
BMI	kg/m^2^	Min-Max, M ± SD	18.90–24.20, 22.08 ± 1.57	19.50–29.00, 23.63 ± 2.75	0.001
	normal weight	*n* (%)	50 (100.00)	37 (74.00)	
	overweight	*n* (%)	0 (0.00)	13 (26.00)	
Level of education	primary	*n* (%)	5 (10.00)	6 (12.00)	0.948
	secondary	*n* (%)	30 (60.00)	29 (58.00)	
	tertiary	*n* (%)	15 (30.00)	15 (30.00)	
Place of residence	village	*n* (%)	17 (34.00)	13 (26.00)	0.538
	Town	*n* (%)	17 (34.00)	16 (32.00)	
	City	*n* (%)	16 (32.00)	21 (42.00)	
Type of job	physical	*n* (%)	13 (26.00)	12 (24.00)	0.927
	mixed	*n* (%)	33 (66.00)	33 (66.00)	
	intellectual	*n* (%)	4 (8.00)	5 (10.00)	
Monthly income per capita in household (ths. PLN)	below 2	*n* (%)	8 (16.00)	6 (12. 00)	0.723
	2–3.5	*n* (%)	30 (60.00)	29 (58.00)	
	over 3.5	*n* (%)	12 (24.00)	15 (30.00)	

^1^ Chi-square test for categorical characteristics or Student’s *t* test for numerical characteristics. BMI—Body mass index, M—Mean, SD—Standard deviation. IU—international unit; ths. PLN—TechShares in Polish Zloty.

**Table 2:** Semen parameters and sex hormones compared between the study group and the control group.

Variable	IU or Category	Parameter	Study Group (N = 50)	Control Group (N = 50)	*p* ^1^
Total sperm count	mln	M ± SD	112.02 ± 52.22	286.46 ± 137.76	<0.001
	below standard (<39)	*n* (%)	2 (4.00)	0 (0.00)	0.153
Sperm count	mln/mL	M ± SD	20.97 ± 4.81	55.16 ± 23.28	<0.001
	below standard (<15)	*n* (%)	6 (12.00)	0 (0.00)	0.012
Semen volume	mL	M ± SD	5.29 ± 1.90	5.37 ± 2.04	0.840
	below standard (<1.5)	*n* (%)	0 (0.00)	0 (0.00)	1.000
Normal morphology	%	M ± SD	3.92 ± 2.28	9.62 ± 5.47	<0.001
	below standard (<4)	*n* (%)	21 (42.00)	11 (22.00)	0.032
Viability	%	M ± SD	65.42 ± 16.51	74.98 ± 15.23	0.003
	below standard (<58)	*n* (%)	21 (42.00)	6 (12.00)	0.001
Progressive motility	%	M ± SD	32.88 ± 7.24	49.48 ± 9.00	<0.001
	below standard (<32)	*n* (%)	23 (46.00)	3 (6.00)	<0.001
FSH	IU/L	M ± SD	5.63 ± 1.79	5.68 ± 2.23	0.905
	above standard (>7.6)	*n* (%)	9 (18.00)	9 (18.00)	1.000
LH	IU/L	M ± SD	5.77 ± 2.10	5.87 ± 2.35	0.810
	above standard (>9.9)	*n* (%)	2 (4.00)	2 (4.00)	1.000
TTE	nmol/L	M ± SD	20.26 ± 5.84	21.28 ± 6.09	0.395
	above standard (>34.72)	*n* (%)	0 (0.00)	0 (0.00)	1.000
DFI	%	M ± SD	28.90 ± 12.78	14.62 ± 8.60	<0.001
	above standard (>30)	*n* (%)	26 (42.00)	3 (6.00)	<0.001
ORP	mV/106 sperm/mL	M ± SD	1.55 ± 0.59	0.79 ± 0.59	<0.001
	above standard (>1.36)	*n* (%)	35 (70.00)	6 (12.00)	<0.001
Microbial pathogen	Positive	*n* (%)	13 (26.00)	5 (10.00)	0.037

^1^ Chi-square test for categorical variables or Student’s *t* test for numerical variables. M—Mean, SD—Standard deviation, FSH—follicle-stimulating hormone, LH—lueinizing hormone, TTE—testosterone, DFI—fragmentation index, ORP—oxidation-reduction potential, IU—international unit.

**Table 3:** Correlations between semen parameters in the study group and the control group.

Variable		Study Group (N = 50)				Control Group (N = 50)			
		MAR IgA (%)	MAR IgG (%)	DFI (%)	ORP (mV/106 Sperm/mL)	MAR IgA (%)	MAR IgG (%)	DFI (%)	ORP (mV/106 Sperm/mL)
Total sperm count (mln)	r	0.031	0.065	−0.524	−0.494	−0.089	−0.139	−0.459	−0.457
	*p*	0.833	0.655	<0.001	<0.001	0.537	0.337	0.001	0.001
Sperm count (mln/mL)	r	0.205	0.232	−0.969	−0.915	0.163	0.180	−0.636	−0.651
	*p*	0.153	0.105	<0.001	<0.001	0.260	0.212	<0.001	<0.001
Semen volume (mL)	r	−0.094	−0.063	−0.037	−0.028	−0.152	−0.191	0.087	0.092
	*p*	0.515	0.665	0.799	0.847	0.293	0.184	0.550	0.526
Normal morphology (%)	r	0.179	0.181	−0.838	−0.895	0.030	0.001	−0.810	−0.806
	*p*	0.213	0.209	<0.001	<0.001	0.834	0.992	<0.001	<0.001
Viability (%)	r	0.181	0.211	−0.981	−0958	−0.163	−0.152	−0.928	−0.898
	*p*	0.209	0.140	<0.001	<0.001	0.258	0.292	<0.001	<0.001
Progressive motility (%)	r	0.191	0.223	−0.962	−0.937	−0.044	0.018	−0.966	−0.961
	*p*	0.185	0.120	<0.001	<0.001	0.761	0.901	<0.001	<0.001

r—Pearson’s correlation coefficient. MAR—mixed antiglobulin reaction, DFI—DNA fragmentation index, ORP—oxidation-reduction potential, Ig—immunoglobulin.

**Table 4:** Correlations between presence of anti-sperm antibodies and abnormal semen parameters and in the study group and the control group.

Variable	Abnormal Interval	Study Group			Control Group		
		Anti-Sperm Antibodies		*p*	Anti-Sperm Antibodies		*p*
		Present (N = 42)	Absent (N = 8)		Present (N = 35)	Absent (N = 15)	
Total sperm count	below standard (<39 mln)	1 (2.38)	1 (12.50)	0.297	n/a	n/a	
Sperm count	below standard (<15 mln/mL)	6 (14.29)	0 (0.00)	0.330	n/a	n/a	
Semen volume	below standard (<1.5 mL)	n/a	n/a		n/a	n/a	
Normal morphology	below standard (<4%)	19 (45.24)	2 (25.00)	0.570	9 (25.71)	2 (13.33)	0.283
Viability	below standard (<58%)	18 (42.86)	3 (37.50)	0.549	6 (17.14)	0 (0.00)	0.102
Progressive motility	below standard (<32%)	20 (47.62)	3 (37.50)	0.711	3 (8.57)	0 (0.00)	0.334
DFI	above standard (>30%)	23 (54.76)	3 (37.50)	0.305	3 (8.57)	0 (0.00)	0.334

Results are presented as N (%). *p* for Fisher’s exact test. n/a—Not applicable because all men had these semen parameters in standards. DFI— DNA fragmentation index.

**Table 5:** Semen parameters, oxidation-reduction potential (ORP) and DNA fragmentation index (DFI) versus microbial pathogen.

Variable	Study Group (N = 50)			Control Group (N = 50)		
	Positive Microbial Pathogen (N = 13)	Negative Microbial Pathogen (N = 37)	*p* ^1^	Positive Microbial Pathogen (N = 5)	Negative Microbial Pathogen (N = 45)	*p* ^1^
Total sperm count (mln), M ± SD	73.65 ± 29.19	125.51 ± 52.05	<0.001	207.60 ± 92.33	295.23 ± 139.90	0.196
Sperm count (mln/mL), M ± SD	15.02 ± 2.97	23.06 ± 3.36	<0.001	32.00 ± 6.56	57.74 ± 23.06	<0.001
Semen volume (mL), M ± SD	4.96 ± 1.90	5.41 ± 1.92	0.523	6.30 ± 2.54	5.27 ± 1.99	0.358
Normal morphology (%), M ± SD	2.77 ± 0.93	8.38 ± 4.03	<0.001	3.00 ± 2.24	10.36 ± 5.23	0.003
Viability (%), M ± SD	46.15 ± 8.77	72.19 ± 12.82	<0.001	42.20 ± 14.38	78.62 ± 10.20	<0.001
Progressive motility (%), M ± SD	23.49 ± 1.91	36.18 ± 5.20	<0.001	30.93 ± 5.80	51.54 ± 6.61	<0.001
ORP (mV/106 sperm/mL), M ± SD	2.13 ± 0.22	1.34 ± 0.54	<0.001	2.14 ± 0.43	0.64 ± 0.37	<0.001
DFI (%), M ± SD	44.92 ± 9.28	23.27 ± 8.28	<0.001	33.80 ± 5.89	12.49 ± 5.72	<0.001

^1^ Mann-Whitney’s U test. M—Mean, SD—Standard deviation. DFI—DNA fragmentation index, ORP—oxidation-reduction potential.

## Data Availability

The data presented in this study are available on request from Artur Wdowiak. The data are not publicly available due to privacy restrictions.
